# Impact of sex differences on outcomes in patients with non-valvular atrial fibrillation undergoing left atrial appendage closure: A single-center experience

**DOI:** 10.7150/ijms.53221

**Published:** 2021-03-03

**Authors:** Mingzhong Zhao, Felix Post, Manuela Muenzel, Cody R. Hou, Thorsten Keil, Jiangtao Yu

**Affiliations:** 1 Department of Cardiology, Helmut-G.-Walther-Klinikum, Lichtenfels, Germany.; 2 Heart Center, Zhengzhou Ninth People's Hospital, Zhengzhou, China.; 3 Clinic for General Internal Medicine and Cardiology, Katholisches Klinikum Koblenz Montabaur, Germany.; 4 University of Minnesota Medical School, Minneapolis, MN, USA.; 5 Department of Anesthesiology, Helmut-G.-Walther-Klinikum, Lichtenfels, Germany.

**Keywords:** non-valvular atrial fibrillation, left atrial appendage, LAA closure, outcomes, sex differences

## Abstract

Female patients affected by non-valvular atrial fibrillation (NVAF) have a higher risk of stroke compared with male patients. Left atrial appendage (LAA) closure has been demonstrated as a reasonable alternative to warfarin therapy for stroke prevention in patients with NVAF. However, the impact of sex-related differences on outcomes in patients undergoing LAA closure (LAAC) remains unclear. Our study investigated the differences in LAAC efficacy and safety endpoints between sexes. 387 consecutive patients undergoing WATCHMAN device implantation were enrolled and stratified by sex. Baseline clinical characteristics, procedural data, severe peri-procedural complications and long-term outcomes were compared between men and women. Measurements of LAA width and depth, device implantation success rate, and the frequency of severe peri-procedural complications were comparable between the two groups. After an average follow-up length of two years post LAAC, no significant differences were observed in the risks for composite thromboembolic events (P = 0.096), major bleeding (P = 0.129), and combined primary (co-primary) efficacy events (P = 0.231) between sexes, but the risk of all-cause death decreased significantly in women compared with men (P = 0.045). After performing propensity matching adjustment for residual confounders, the sex-related differences in the cumulative ratio of freedom from all-cause death did not reach statistical significance (P = 0.062), as was also observed with the cumulative ratio of freedom from composite thromboembolic events (P = 0.104), major bleeding (P = 0.134), and co-primary efficacy events (P = 0.241). The observed annual rate of thromboembolic events was significantly decreased by 67.1% (P < 0.01) and 52.5% (P < 0.05) and the observed annual rate of bleeding was reduced by 33.6% (P < 0.05) and 43.5% (P < 0.05) in men and women when compared with the predicted risk based on CHA_2_DS_2_VASc score and HAS-BLED score, respectively. LAAC can be considered as an effective and safe strategy in preventing thromboembolic events and decreasing bleeding risks in NVAF patients, regardless of sex. LAAC appears to normalize the sex-specific differences in NVAF patients both in terms of safety and efficacy.

## Introduction

Atrial fibrillation (AF) is the most common type of cardiac arrhythmia worldwide, among which non-valvular atrial fibrillation (NVAF) accounts for the most cases of AF in developed countries 1. Aging and chronic heart diseases are among the factors responsible for the rising prevalence of AF 2. A total of 1%-2% of the population in North America and Europe suffer from AF. In China, the prevalence of AF ranges from 0.37% to 3.75% 3. NVAF is associated with an approximately two to seven-fold increase in the risk of stroke and a twofold increase in the risk of death. Cardioembolic stroke and systemic embolism are the most serious complications in patients with NVAF 4.

According to European and US guidelines, anticoagulation therapy applying either warfarin or non-vitamin K antagonist oral anticoagulants (NOACs) are recommended for AF treatment 5-6. Although anticoagulation is an effective treatment for stroke prevention, warfarin administration shows some limitations in clinical practice, including interactions with multiple drugs and food, increased risk of bleeding and a narrow therapeutic window requiring regular blood monitoring of international normalized ratio (INR) 7. These shortcomings attenuate the efficacy of warfarin and patient adherence. Although NOACs do not require coagulation monitoring and demonstrate similar or even better clinical benefits for stroke prevention and risk reduction of major bleeding compared with warfarin, they also face the risk of bleeding and recurrent cardioembolic stroke 8-9.

More than 90% of heart thrombi that cause cardioembolic stroke and systemic embolism in NVAF patients originate in the left atrial appendage (LAA) 10. In recent years, a new intervention strategy, LAA closure (LAAC), has emerged as a viable treatment alternative to warfarin for stroke prophylaxis in NVAF. The 5 year outcomes of the PREVAIL trial, combined with the PROTECT AF trial, showed that LAAC was non-inferior in preventing post-implant stroke or systemic embolism to warfarin with additional reductions in the risk of hemorrhagic and/or disabling stroke as well as cardiovascular death 11. It was reported that female patients with AF were at higher risk for stroke compared with male patients, even with prescription of warfarin 12-13. Women who received continuous anticoagulation treatment after AF ablation also experienced longer hospital stays and more bleeding events than men 14. However, the influence of sex-associated differences on clinical outcomes in NVAF patients undergoing LAAC has not yet been elucidated. Therefore, we sought to investigate the effect of sex differences on the efficacy and safety of LAAC in NVAF patients.

## Methods

### Study population

From February 2012 to September 2018, 387 consecutive NVAF patients with high bleeding risk for oral anticoagulation underwent percutaneous LAAC procedures using the WATCHMAN™ (WM) occluder (Boston Scientific, Marlborough, MA, USA) at Helmut-G.-Walther-Klinikum, Lichtenfels, Germany. Individuals enrolled in the study met the following inclusion criteria: NVAF patients with high risk for stroke or thromboembolic events [CHA_2_DS_2_VASc (Congestive heart failure, Hypertension, Age ≥ 75 years, Diabetes mellitus, Stroke/transient ischemic attack, Vascular disease, Age 65 to 74 years, Sex category) scores > 2 or presence of previous history of stroke, transient ischemic attacks (TIA), or peripheral embolism], or with contraindication for long-term usage of oral anticoagulation (OAC) therapy [high risk for bleeding with HAS-BLED (Hypertension, Abnormal renal / liver function, Stroke, Bleeding history or predisposition, Labile INR, Elderly, Drugs / alcohol concomitantly) scores ≥ 3, positive history of hemorrhagic stroke or major bleeding, or unstable INR] or OAC refusal. All subjects gave written informed consent. Patients with severe consumptive disease with life expectancy shorter than 1 year or echocardiographic evidence of thrombus in left atria / left atrial appendage were excluded from the study. All patients who underwent LAAC were categorized into men and women groups. Data of demographic and clinical characteristics, procedure data, severe peri-procedural complications and long-term follow-up outcomes were collected and analyzed. This study complies with the Declaration of Helsinki and was approved by the Ethics Committee at Helmut-G.-Walther-Klinikum, Lichtenfels, Germany.

### LAAC procedure

The implantation procedure has been described in a previous study 11. Briefly, device implantation for LAA was performed under general anesthesia with intra-procedural transesophageal echocardiography (TEE) and fluoroscopy guidance. After successfully puncturing the atrial septum, unfractionated heparin was administered intravenously at a dose of 70-100 IU / Kg, to maintain > 250 seconds of activated clotting time (ACT) during the procedure. The optimal device size was determined according to TEE and X-ray angiograms and was based on the recommended compression ratio in relation to the size of the LAA. All implantations of the device met PASS criteria (position, anchor, size, and seal) before release of the device. Successful closure of LAA was confirmed by TEE defined as no or minimal leak flow (gap < 5 mm).

Patients were required to stay in the hospital overnight after the implantation; those without significant pericardial effusion, procedure-related major bleeding, or other severe peri-procedure complications were discharged the next day.

### Anti-thrombotic regimen post-procedure

After the procedure, an anti-thrombotic regimen was initiated to give time for device endothelialization. (1) During the first 45 days post-procedure, subjects were treated with either warfarin or NOACs combined with aspirin; combinations of enoxaparin and aspirin were prescribed in those with contraindications for warfarin. (2) At the 45-day visit, anticoagulants were discontinued and the patients were given dual antiplatelet therapy (aspirin and clopidogrel) if TEE showed adequate closure of the LAA with no apparent peri-device leak (< 5 mm in width) or device-related thrombus (DRT). (3) After the 6-month visit, patients were treated with aspirin alone indefinitely if TEE follow-up indicated neither DRT nor peri-device flow ≥ 3 mm. If inadequate peri-device flow was obtained or a thrombus was detected, the anticoagulation regimen therapy was restarted with warfarin or NOACs and aspirin until an adequate seal or complete disappearance of the thrombus was confirmed by repeat TEE exam.

### Follow-up

Baseline characteristics, procedural data, severe periprocedural complications and clinical outcomes of all patients were recorded. The TEE follow-up was performed at least twice at 45 days and 6 months after procedure. The clinical outcomes were collected by outpatient visit or telephone follow-up.

The endpoints of the study were defined as: (1) implantation success rate; (2) severe peri-procedural complications within 7 days defined as stroke, TIA, other systemic embolism, DRT, major bleeding (intracranial hemorrhage/gastrointestinal bleeding/other major bleeding requiring invasive treatment or blood transfusion), pericardial effusion/cardiac tamponade, severe vascular complications or device-related death; (3) major adverse events during long-term follow-up, defined as any single adverse event including stroke, TIA other systemic embolism, DRT, cerebral hemorrhage, gastrointestinal bleeding, other major bleeding, cardiovascular death, non-cardiovascular death, or a composite of any of these events which mainly contained composite thromboembolic events (ischemic stroke/TIA/systemic embolism), major bleeding events (cerebral hemorrhage/gastrointestinal bleeding/other bleeding events), all-cause death (cardiovascular death/non-cardiovascular death), and combined primary (co-primary) efficacy events (composite thromboembolic events/all-cause death).

### Risk assessment of thromboembolic and bleeding events

The observed annual rates of thromboembolic (stroke, TIA and systemic embolism) and bleeding events were calculated as follows: the total numbers of thromboembolic or bleeding events during follow-up were divided by the total patient-years of follow-up and were multiplied by 100 to get the observed annual rate of thromboembolic or bleeding events, which was expressed with events/per 100 patient-years, respectively. As thromboembolic events increase with CHA_2_DS_2_-VASc score, the expected rate of thromboembolic events was evaluated quantitatively according to CHA_2_DS_2_-VASc score in AF patients with no prescription of warfarin throughout follow up 15. Similarly, as bleeding risks increase with HAS-BLED score, the expected rate of bleeding events was assessed based on HAS-BLED score in AF patients taking warfarin 16. The expected annual rates of thromboembolic and bleeding events in a group were calculated as the sum of the expected annual thromboembolism rates/total numbers of patients and the sum of the expected annual bleeding rates/total numbers of patients, respectively.

### Statistical analysis

Results are presented as number of patients and percentages for categorical variables. Differences between groups were tested using the Fisher's exact test. Continuous variables are expressed as mean ± standard deviation (SD) or as medians with interquartile ranges (25^th^ and 75^th^ percentiles). Continuous variables were compared using the independent samples *t-*test. We estimated hazard ratios (HR) and their 95% confidence intervals (CI) by Cox proportional hazard models before and after adjustment for potential confounding factors with propensity score-matching analysis. We used Kaplan-Meier curves to describe the cumulative ratio of freedom from the combined adverse events. A log-rank test was used to evaluate the differences of the cumulated ratios between men and women. To evaluate the efficacy of LAAC for the prevention of thromboembolic events and reduction of bleeding events, the comparisons between the actual event rate and the predicted event rate based on the CHA_2_DS_2_-VASc score and HAS-BLED score were made both in men and women groups respectively with a Chi-square test. A P-value < 0.05 was judged to be significant. Analyses were performed using SPSS version 22.0 (SPSS Inc., Chicago, Illinois).

## Results

### Baseline demographic and clinical characteristics

In 387 patients with NVAF, WM device implantation was unsuccessful in six patients due to unsuitable LAA anatomy. They were followed by successful procedures with the Amplatzer Cardiac Plug device (St. Jude Medical, Golden Valley, MN). WM device implantation attempts were halted in 4 cases due to 3 cases of cardiac tamponade and 1 case of repeated device-related thrombus. Of the 3 cases with cardiac tamponade, 2 cases were treated with a conservative strategy (timely pericardial puncture and blood transfusion); the other case required surgical intervention because of unstable hemodynamics despite timely pericardial puncture. The other 1 case of device-related thrombus was recurrent despite attempting to dissolve the thrombus by adding the dose of heparin and using a tirofiban (platelet glycoprotein IIb/IIIa receptor antagonist). Therefore, device placements were abandoned in the four patients. Thus, 377 (97.4%) NVAF patients successfully received WM device implantations. There was no significant difference of procedural success rate between men and women (97.6% vs 96.8%, P = 0.156).

Table 1 shows the detailed results of baseline demographics and clinical characteristics of the participants. The percentage of patients with previous stroke/TIA, previous major bleeding, or liver dysfunction were comparable between genders. The HAS-BLED score in women was similar to that in men. However, women were older and more often had hypertension, diabetes mellitus, impaired renal function, and paroxysmal/persistent atrial fibrillation as well as higher mean score of CHA_2_DS_2_-VASc. They were less likely to have coronary heart disease (CHD) and chronic heart failure. There was no sex preference in the prescription of antithrombotic agents at baseline (Table 1).

### Procedural data

All patients were examined by TEE. There were no pronounced differences in LAA width, LAA depth, and WM device sizes between the two groups. During the procedure, the percentage of patients with a recapture of the device was higher in men compared with women. A leakage of >5 mm around the device was not observed at all. No significant difference in peri-device flow within ≤ 5 mm was detected between the two groups. Except for the volume of contrast dye used (mL) which was lower in the female group, procedural parameters such as fluoroscopy time (min) and X ray-dose [mGy cm^2^] were comparable between the two groups (Table 2).

### Severe peri-procedural complications

Severe procedure-related complications within 7 days occurred at a rate of 4.0%. Of the 377 patients, who underwent LAAC successfully, there were one stroke event in the male group, 4 device-associated thrombi (3 events in male group and 1 event in the female group), 2 major bleeding events (1 event in each group), 3 pericardial effusions/cardiac tamponades (2 in the male group and 1 in the female group), 5 severe vascular complications (3 in the male group and 2 in the female group). There was no device-related death within 7 days of the peri-procedural period. No statistical differences were observed for these severe peri-procedural complications between the two groups (Table 3).

### Long-term follow-up outcomes

Clinical follow-ups were performed for all participants to monitor the major adverse events through outpatient service or telephone visits. The average follow-up time for the cohort was 779.8 ± 537.7 days (men: 801.2 ± 561.1 days; women: 784.3 ± 517.4 days). No significant differences were found in average follow-up time and TEE examination rate between male and female groups.

**Figure 1:**
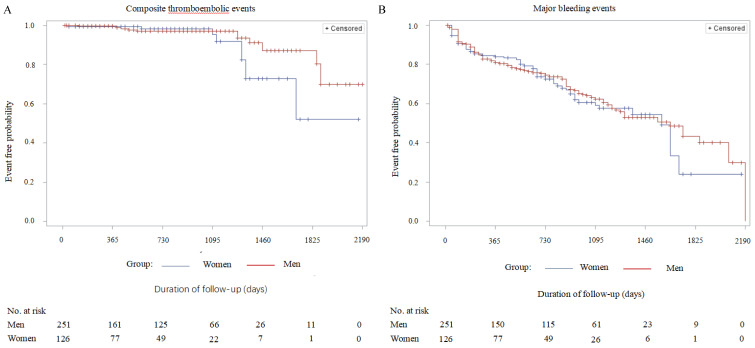

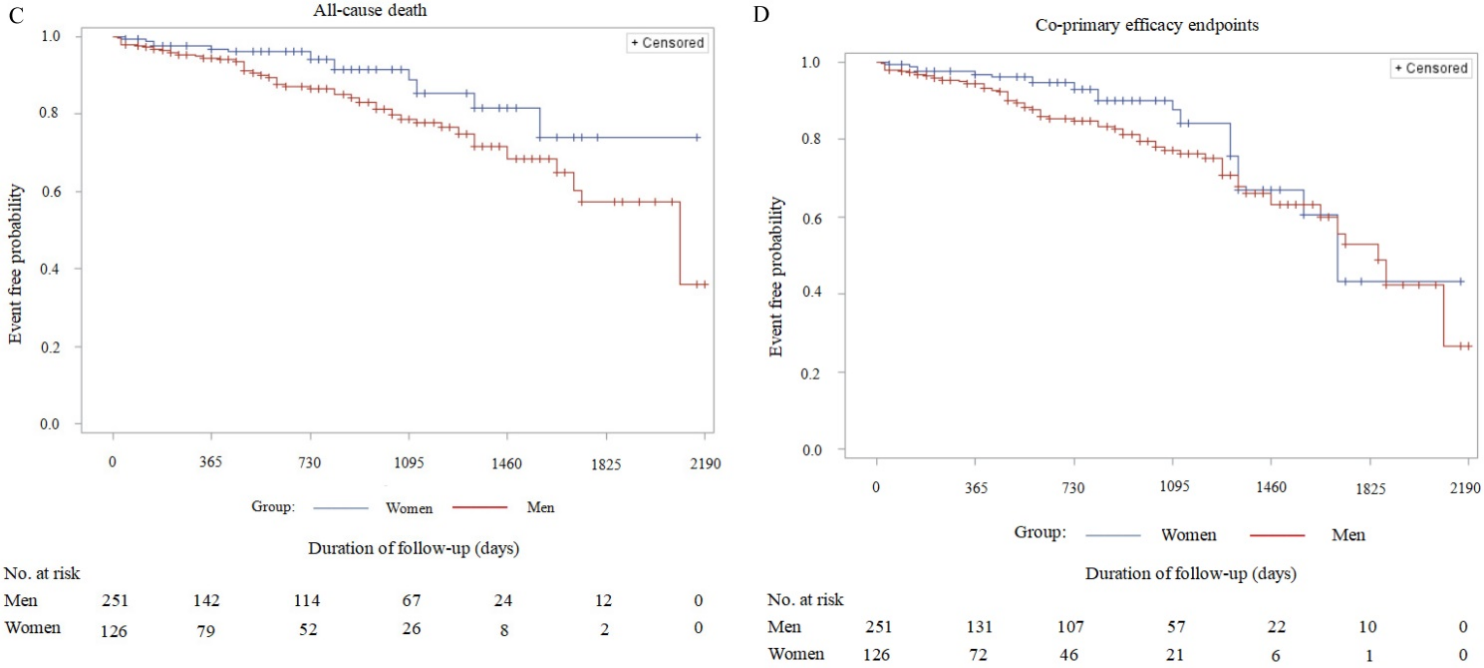
Kaplan-Meier survival curves of the cumulative ratio of freedom from major adverse events (men vs. women, after adjustment for the residual confounders): A: Cumulative ratio of freedom from composite thromboembolic events - stroke/TIA /systemic embolism; Log Rank (Mantel-Cox), P=0.104. B: Cumulative ratio of freedom from major bleeding events - cerebral hemorrhage/gastrointestinal bleeding/other bleeding; Log Rank (Mantel-Cox), P=0.134. C: Cumulative ratio of freedom from all-cause death - cardiovascular death/non-cardiovascular death; Log Rank (Mantel-Cox), P=0.062. D: Cumulative ratio of freedom from the combined primary efficacy events - stroke/TIA/systemic embolism/all-cause death; Log Rank (Mantel-Cox), P=0.241.

Detailed information about major adverse events during the period of long-term follow-up is presented in Table 4. The rate of all-cause mortality (cardiovascular death/non-cardiovascular death) in the female group was significantly lower compared with the male group (7.94% vs 18.33, P = 0.045). However, the incidences of predefined single adverse events such as ischemic stroke, TIA, systemic embolism, DRT, cerebral hemorrhage, gastrointestinal bleeding, other major bleedings, cardiovascular and non-cardiovascular death were not statistically different between the groups. Combined adverse events, such as composite thromboembolic events (P = 0.096), major bleeding (P = 0.129), and co-primary efficacy events (P = 0.231) were also comparable (Table 4). After adjustment for residual confounders, such as age, hypertension, diabetes, CHD, chronic heart failure, impaired renal function and types of atrial fibrillation according to the propensity score matching, Kaplan-Meier survival curve analysis demonstrated no statistical difference in the cumulative ratio of freedom from all-cause death in women compared with men (P = 0.062). Meanwhile, the other three variables covering cumulative ratios of freedom from composite thromboembolic events (P = 0.104), major bleedings (P = 0.134), and co-primary efficacy events (P = 0.241) also were not significantly different between the two groups (Figure 1). The baseline characteristics of these confounders mentioned above before and after adjustment with propensity score matching are shown in Table S1 as an online data supplement.

### Comparative analysis of the observed annual rate of composite thromboembolic events or major bleeding events and the predicted risk

**Figure 2:**
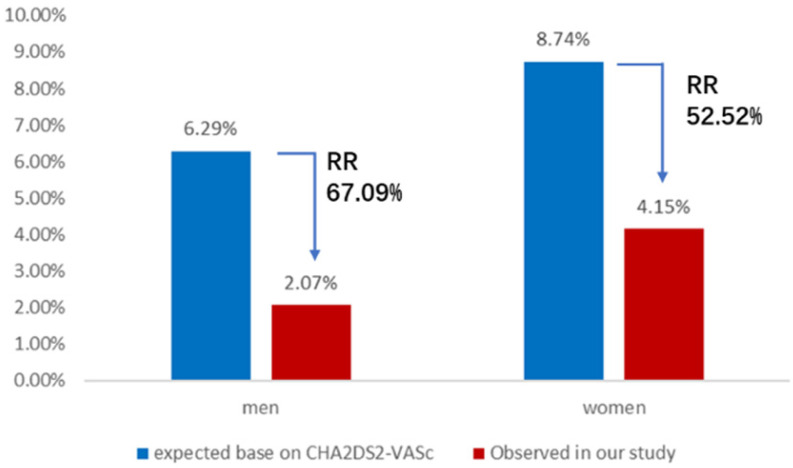
Annual rate of composite thromboembolic events. Observed annual rate of composite thromboembolic events, expected annual rate of composite thromboembolic events based on the CHA_2_DS_2_-VASc scores, and the relative risk reduction for men or women group. RR: relative risk.

**Figure 3:**
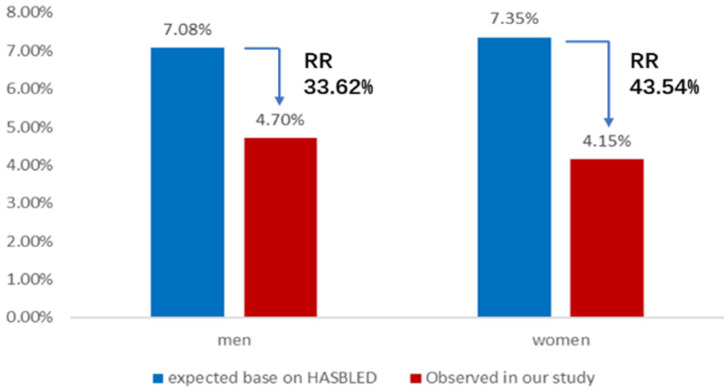
Annual rate of major bleeding events. Observed annual rate of major bleeding events, expected annual rate of major bleeding events based on the HAS-BLED score, and the relative risk reduction for men or women group. RR: relative risk.

The expected annual rate of composite thromboembolic events in AF patients without the anticoagulant treatment based on CHA_2_DS_2_-VASc score is 6.29 per 100 patient-years in the male group and 8.74 per 100 patient-years in the female group. Nevertheless, the observed annual rate of composite thromboembolic events was 2.07 and 4.15 per 100 patient-years in men and women, respectively. The results of comparative analysis indicated a 67.1% relative risk reduction for composite thromboembolic events of men (X^2^ = 12.58, P < 0.01) and a 52.5% relative risk reduction for composite thromboembolic events of women (X^2^ = 4.22, P < 0.05) (Figure 2). Meanwhile, the expected annual rate of major bleeding in patients taking anticoagulant therapy based on HAS-BLED score is 7.08 per 100 patient-years in the male group and 7.35 per 100 patient-years in the female group, whereas the observed annual rate of major bleeding in this study was 4.70 and 4.15 per 100 patient-years in men and women, respectively. These represented a 33.6% relative risk reduction for major bleeding events of men (X^2^ = 3.85, P < 0.05) and a 43.5% relative risk reduction for major bleeding events of women (X^2^ = 3.92, P < 0.05) (Figure 3).

## Discussion

Although NVAF is an age-related disease in both men and women, many studies suggest that different risk factors for cardiovascular disease exist between male and female AF patients 17,18. In this study, advanced age, hypertension, diabetes and impaired renal function were more often observed in women, which corresponded to the findings from previous studies 17,19. Women also had a higher mean CHA_2_DS_2_-VASc score than men. However, other comorbidities such as CHD, heart failure were less likely to occur in women. Advanced age, hypertension and diabetes are important risk factors for cardioembolic and major bleeding events, and impaired renal function seems to worsen clinical outcomes in AF patients 20. In fact, higher CHA_2_DS_2_-VASc score predicted a higher risk of stroke or systemic embolism. Therefore, female AF patients with higher CHA_2_DS_2_-VASc score were considered to have an increased risk of adverse cardiovascular events as compared to males according to the baseline clinical characteristics of the cohort. Other studies also supported this perspective in which female AF patients had a worse outcome and higher rates of readmission and bleeding complications relative to men 21,22. In NVAF, the majority of thrombi leading to cardioembolic events derive from the LAA. Increasing clinical data, including our previous study, demonstrated that LAAC was a valid alternative to oral anticoagulation therapy for preventing from stroke or systemic embolisms 23-25. However, the effects of sex-related differences on the clinical outcomes of LAAC in NVAF patients remain unknown.

Regarding sex-related differences in LAA sizes, Boucebci S et al. reported that men had wider and longer LAAs compared with women in normal conditions in cardiac CT scans 26. LAA-orifice diameter has previously been considered to be correlated with left atrial volume and to be smaller in paroxysmal AF patients when compared with non-paroxysmal AF patients 27. In our study, the LAA width and length (depth) were comparable, and the successful implantation rates of device were similar between sexes.

Of the 377 AF patients receiving LAAC, the incidence of severe complications within the 7-day peri-procedural period was very low at 4.0% without any procedure-related death. This periprocedural adverse event rate was similar to that of a previous study which reported that severe procedure-related complications within the first 7 days occurred in a rate of 4.2% 28. Moreover, no significant differences were presented for the procedure-related adverse events between men and women in our study. This implies the LAAC procedure is safe and feasible both in male and female AF patients.

Regarding sex-associated outcome differences in anticoagulant treatment in patients with AF, warfarin did not resolve the effect of sex difference on clinical outcomes, as female patients also exhibited higher risks of stroke and embolism while taking warfarin than male patients did 29. Unlike warfarin, NOACs seemed to eliminate the discrepancy of prognosis between sexes 13. However, does LAAC have the same effect as NOACs? The results of our average two year follow up indicated that there were no significant differences between genders in the single adverse events or in the combined adverse events, such as composite thromboembolic events, major bleeding, and co-primary efficacy events, except for the events of all-cause death. In our study, female patients demonstrated a statistically significantly lower risk in all-cause mortality compared with male patients before adjustment of confounding factors. However, after carrying out propensity score-matching analysis by adjusting the relevant confounding factors, such as age, hypertension, diabetes, CHD, chronic heart failure, impaired renal function, and types of atrial fibrillation, the impact of sex-related differences on the cumulative ratio of freedom from all-cause death disappeared. Moreover, the cumulative ratios of freedom from composite thromboembolic events, major bleeding, and co-primary efficacy events, respectively, did not differ between sexes. Our results indicate that we have no sufficient evidence to support that female AF patients suffered from more complications and worse long-term outcomes post LAAC procedure compared with men. Additionally, we also do not have enough evidence to confirm the sex-related differences in long-term effectiveness and safety of LAAC in AF patients. LAAC strategy seems to normalize the sex-specific differences both in terms of efficacy and safety. Interestingly, we noticed that there was a numerically higher trend for cumulative ratio of freedom from all-cause death in women compared to men with a borderline significant difference (P=0.062, no statistical significance, but close to significance) after performing propensity matching analysis. Therefore, we wonder whether or not female AF patients may benefit more from LAAC strategy in reducing the risk of all-cause death compared to men. This would need to be evaluated in larger scale trials.

Recently, device-related thrombus (DRT) has been disputed as to whether it is significantly associated with later stroke or systemic embolism 30,31. After an average 2-year follow up of the total cohort, DRT was present in 5.3% of patients with the WATCHMAN™ device. The incidence of DRT was slightly higher than the 3.7% DRT in the EWOLUTION trial 32, and comparable to the PROTECT-AF trial (DRT 5%) 33, but lower than that in the French RELAXAO registry study (DRT up to 7.2%) 31. Many factors, such as device, procedure and patient's conditions may affect the occurrence of DRT. However, our subgroup analysis indicated no significant difference in the incidence of DRT between men and women.

In our study, the observed annual rate of composite thromboembolic events was 2.07% in men and 4.15% in women, leading to a 67.9% relative risk reduction in men and a 52.52% relative risk reduction in women with statistical significance, compared with the expected annual risk based on CHA_2_DS_2_-VASc scores, respectively. The results were similar to those reported from ACP2 study which presented 69% relative risk reduction in the observed rates of total stroke, compared with the predicted risk, in NVAF patients who underwent LAAC with the WATCHMAN™ device 34. Meanwhile, the observed annual major bleeding rate showed a 33.62% relative risk reduction in men and a 43.54% relative risk reduction in women, compared with the expected annual bleeding rate based on HAS-BLED score, respectively. So, our results demonstrated that both male and female AF patients benefited significantly from LAAC in preventing thromboembolic events and decreasing major bleeding risk. In line with previous research results 35,36, these conclusions of long-term follow-up provided stronger evidence to further support that LAAC is a safe and effective strategy for stroke prevention and declining bleeding risk in NVAF patients.

This study has several limitations. First, the study is a retrospective analysis conducted in a single center without randomization and control group. Although our results were comparable to those from other clinical trials, the conclusions must still be interpreted with caution in the absence of a matched control arm. Second, the prescription rates of antithrombotic drugs such as warfarin, antiplatelet agents and NOACs during the periprocedural period varied between patients who admitted in the earlier and later stages, which might have influenced the clinical outcomes. Third, TEE measurements and interpretation were performed by operators without independent image adjudication. Finally, the relatively low number of patients included in this study did not allow us to definitely determine or exclude the possibility that women may have a better prognosis than men after LAAC. In spite of these limitations, the present study provided the single-center experience of 377 consecutive patients undergoing LAAC and the data might reflect “real-world” clinical practice.

In conclusion, the study presented no statistical differences in LAAC procedural success rates and severe peri-procedural complications within 7-days after the procedure between male and female AF patients. Long-term follow-up demonstrated no significant differences in the risks for composite thromboembolic events, major bleeding events and co-primary efficacy events, nor in the cumulative ratios for freedom of composite thromboembolic events, major bleeding, all-cause death, and co-primary efficacy events between sexes. Our real-world experience showed that LAAC could not only prevent thromboembolisms and decrease bleeding risks compared with the predicted risk both in men and women, respectively, but also attenuate the influence of sex-related differences on clinical outcomes in AF patients. LAAC may be an optimal choice in decreasing the risks of cardioembolic and bleeding events in AF patients, regardless of sex.

## Supplementary Material

Supplementary table S1.

## Figures and Tables

**Table 1:** Baseline demographic and clinical characteristics

Major adverse events	All (n=377)	Men (n=251)	Women (n=126)	*P* value
Age, years (mean ± SD)	76.5 ± 8.3	74.1 ± 8.3	77.5 ± 6.2	0.016
≥75 years, n (%)	225 (59.7)	138 (55.0)	87 (69.1)	< 0.0001
Hypertension, n (%)	303 (80.4)	198 (78.9)	105 (83.3)	0.036
Diabetes mellitus, n (%)	103 (27.3)	66 (26.3)	37 (29.4)	< 0.0001
CHD, n (%)	184 (48.8)	137 (54.6)	47 (37.3)	< 0.0001
Chronic heart failure, n (%)	56 (14.9)	44 (17.5)	15 (11.9)	0.001
Previous stroke/TIA, n (%)	82 (21.8)	51 (20.3)	31 (24.6)	0.079
Previous major bleeding, n (%)	137 (36.3)	88 (35.1)	49 (38.9)	0.154
Liver dysfunction, n (%)	49 (13.0)	36 (14.3)	13 (10.3)	0.245
Impaired renal function, n (%)	172 (45.6)	104 (41.4)	68 (54.0)	0.044
CHA2DS2-VASc score (mean ± SD)	3.8 ± 1.5	3.6 ± 1.3	4.3 ± 1.6	< 0.001
HAS-BLED score (mean ± SD)	3.5 ± 1.0	3.6 ± 1.1	3.5 ± 1.0	0.459
AF, paroxysmal/persistent, n (%)	128 (34.0)	75 (29.9)	53 (42.1)	0.013
AF, permanent, n (%)	249 (66.1)	176 (70.1)	73 (57.9)	0.013
Antithrombotic regimen at baseline, n (%)				
Single antiplatelet agent	132 (35.0)	82 (32.7)	50 (39.7)	0.118
Dual-antiplatelet agent	13 (3.5)	9 (3.6)	4 (3.2)	0.339
Oral warfarin	67 (17.8)	46 (18.3)	21 (16.7)	0.392
Oral NOACs	27 (7.2)	19 (7.6)	8 (6.4)	0.401
Parenteral anticoagulant	124 (32.9)	85 (33.9)	39 (31.0)	0.371
No antithrombotic therapy	14 (3.7)	10 (4.0)	4 (3.2 )	0.294

Categorical variables are expressed as frequencies (n) and percentages (%). Continuous data are reported as means and standard deviation. CHD: coronary heart disease; TIA: transient ischemic attack; AF: atrial fibrillation; NOACs: non-vitamin K antagonist oral anticoagulants.

**Table 2:** Procedural data

Major adverse events	All (n=377)	Men (n=251)	Women (n=126)	*P* value
LAA width (mm)	19.8 ± 3.4	19.9 ± 2.4	19.4 ± 3.1	0.644
LAA depth (mm)	27.6 ± 2.7	28.2 ± 3.5	26.0 ± 3.9	0.951
**Device size (mm)**	25.2 ± 3.1	25.4 ± 3.2	24.7 ± 3.0	0.177
21 mm, n (%)	72 (19.1)	45 (17.9)	27 (21.4)	
24 mm, n (%)	150 (39.8)	96 (38.2)	54 (42.8)	
27 mm, n (%)	108 (28.6)	78 (31.1)	30 (23.8)	
30 mm, n (%)	28 (6.6)	16 (6.4)	12 (9.5)	
33 mm, n (%)	19 (5.0)	16 (6.4)	3 (2.3)	
Device size change, n (%)	24 (6.3)	19 (7.5)	5 (3.9)	0.177
Recapture, n (%)	131 (34.7)	96 (38.2)	35 (27.7)	0.044
**Gap, n (%)**	10 (2.6)	7 (2.7)	3 (2.3)	0.816
<3 mm	9	6	3	
3-5 mm	1	1	0	
>5 mm	0	0	0	
Contrast (ml), median (IQR)	33.5 (21;55)	40 (24;61)	30 (23;57)	0.016
Fluoroscopy time (min), median (IQR)	7 (3;11)	7 (4;10)	7 (3;10)	0.325
X ray-dose [mGy*cm^2^] ], median (IQR)	5192 (3421;6357)	5487 (3249;6842)	4310 (2847;5916)	0.505

LAA: left atrial appendage; IQR: interquartile range.

**Table 3:** Severe complications in the peri-procedure period within 7 days

Major adverse events	All (n=377)	Men (n=251)	Women (n=126)	*P* value
Stroke, n (%)	1 (0.3)	1 (0.4)	0 (0)	1.000
TIA, n (%)	0 (0)	0 (0)	0 (0)	1.000
Other thromboembolism events, n (%)	0 (0)	0 (0)	0 (0)	1.000
Device-related thrombus, n (%)	4 (1.1)	3 (1.2)	1 (0.8)	0.325
Major bleedings, n (%)	2 (0.5)	1 (0.4)	1 (0.8)	0.451
Pericardial effusion/tamponade, n (%)	3 (0.8)	2 (0.8)	1 (0.8)	1.000
Severe vascular complication, n (%)	5 (1.3)	3 (1.2)	2 (1.6)	1.000
Device-related death, n (%)	0 (0)	0 (0)	0 (0)	1.000
Total, n (%)	15 (4.0)	10 (4.0)	5 (4.0)	1.000

Categorical variables are expressed as frequencies (n) and percentages (%). TIA: transient ischemic attack.

**Table 4:** Follow-up data

Major adverse events	All (n=377)	Men (n=251)	Women (n=126)	*P* value
Composite thromboembolic events, n (%)	21 (5.6)	10 (4.0)	11 (8.7)	0.096
Ischemic stroke, n (%)	12 (3.2)	6 (2.4)	6 (4.8)	0.463
TIA, n (%)	9 (2.4)	4 (1.6)	5 (4.0)	0.398
Other systemic embolism, n (%)	0 (0)	0 (0)	0 (0)	1.000
DRT, n (%)	20 (5.3)	15 (6.0)	5 (4.0)	0.227
Residual leak WM > 5 mm, n (%)	2 (0.5)	2 (0.8)	0 (0)	0.241
Major bleedings, n (%)	35 (9.3)	25 (10.0)	10 (7.9 )	0.129
Cerebral hemorrhage, n (%)	4 (1.1)	3 (1.2)	1 (0.8)	0.741
Gastrointestinal bleeding, n (%)	23 (6.1)	16 (6.4)	7 (5.6)	0.794
Other major bleeding, n (%)	8 (2.1)	5 (2.0)	3 (2.4)	0.836
All-cause death, n (%)	56 (14.9)	46 (18.3)	10 (7.9)	0.045
Cardiovascular death, n (%)	14 (3.7)	10 (4.0)	4 (3.2)	0.902
Non-cardiovascular death, n (%)	42 (11.1)	36 (14.3)	6 (4.8)	0.068
Combined primary efficacy events, n (%)	71 (18.8)	50 (19.9)	21 (16.7)	0.231

Categorical variables are expressed as frequencies (n) and percentages (%). Data are presented as number of events with cumulative incidences. Cumulative incidences were estimated by the Kaplan-Meier method. TIA: transient ischemic attack; DRT: device-relative thrombus; WM: WATCHMAN.
