# Supplementary material for: Impact of sex differences on outcomes in patients with non-valvular atrial fibrillation undergoing left atrial appendage closure: A single-center experience
Source: Int J Med Sci. 2021 Mar 3;18(9):1990–8. doi: 10.7150/ijms.53221 (PMC8040400; doi:10.7150/ijms.53221)
Supplement: Supplementary file 1 — Supplementary table S1. [file ijmsv18p1990s1.pdf]

Table S1 The baseline characteristics of confounders before and after adjustment with propensity score matching

| Variables | All<br>N=377 | Men<br>n=251 | Women<br>n=126 | P value |
|---|---|---|---|---|
| Before adjustment with propensity score matching | | | | |
| ≥75 years, n (%) | 225(59.7) | 138(55.0) | 87 (69.1) | < 0.0001 |
| Hypertension, n (%) | 303(80.4) | 198(78.9) | 105(83.3) | 0.036 |
| Diabetes mellitus, n (%) | 103(27.3) | 66(26.3) | 37(29.4) | < 0.0001 |
| CHD, n (%) | 184(48.8) | 137(54.6) | 47(37.3) | < 0.0001 |
| Chronic heart failure, n (%) | 56(14.9) | 44(17.5) | 15 (11.9) | 0.001 |
| Impaired renal function, n (%) | 172(45.6) | 104(41.4) | 68(54.0) | 0.044 |
| AF, paroxysmal / persistent, n (%) | 128(34.0) | 75(29.9) | 53(42.1) | 0.013 |
| AF, permanent, n (%) | 249(66.1) | 176(70.1) | 73(57.9) | 0.013 |
| After adjustment with propensity score matching | | | | |
| Age, ≥75 years, n (%) | 225 (59.7) | 150 (59.8) | 75 (59.3) | 0.923 |
| Hypertension, n (%) | 299 (79.3) | 201 (80.1) | 98 (77.6) | 0.568 |
| Diabetes mellitus, n (%) | 98 (26.0) | 67 (26.8) | 31 (24.5) | 0.640 |
| CHD, n (%) | 183 (48.5) | 122 (48.7) | 61 (48.1) | 0.920 |
| Chronic heart failure, n (%) | 61 (16.3) | 41 (16.4) | 20 (16.1) | 0.946 |
| Impaired renal function, n (%) | 171(45.4) | 116(46.2) | 55(43.7) | 0.687 |
| AF, paroxysmal / persistent, n (%) | 126(33.4) | 85(33.9) | 41(32.5) | 0.975 |
| AF, permanent, n (%) | 245(65.0) | 163(64.9) | 82(65.1) | 0.943 |

Categorical variables are expressed as frequencies (n) and percentages (%).
CHD = coronary heart disease; AF = atrial fibrillation.
